# A Systematic Review of Recent Clinical Practice Guidelines on the Diagnosis, Assessment and Management of Hypertension

**DOI:** 10.1371/journal.pone.0053744

**Published:** 2013-01-17

**Authors:** Lubna A. Al-Ansary, Andrea C. Tricco, Yaser Adi, Ghada Bawazeer, Laure Perrier, Mohammed Al-Ghonaim, Nada AlYousefi, Mariam Tashkandi, Sharon E. Straus

**Affiliations:** 1 Department of Family and Community Medicine, College of Medicine, King Saud University, Riyadh, Saudi Arabia; 2 Shaikh Bahamdan's Research Chair for Evidence-Based Health Care and Knowledge Translation, College of Medicine, King Saud University, Riyadh, Saudi Arabia; 3 Li Ka Shing Knowledge Institute of St Michael's Hospital, Toronto, Ontario, Canada; 4 College of Pharmacy, King Saud University, Riyadh, Saudi Arabia; 5 Prince Salman Bin Abdulaziz Chair for Kidney Disease, Department of Internal Medicine, College of Medicine, King Saud University, Riyadh, Saudi Arabia; 6 Applied Health Research Center, St. Michael's Hospital, Toronto, Ontario, Canada; 7 Division of Geriatrics, University of Toronto, Toronto, Ontario, Canada; University of Washington, United States of America

## Abstract

**Background:**

Despite the availability of clinical practice guidelines (CPGs), optimal hypertension control is not achieved in many parts of the world; one of the challenges is the volume of guidelines on this topic and their variable quality. To systematically review the quality, methodology, and consistency of recommendations of recently-developed national CPGs on the diagnosis, assessment and the management of hypertension.

**Methodology/Principal Findings:**

MEDLINE, EMBASE, guidelines' websites and Google were searched for CPGs written in English on the general management of hypertension in any clinical setting published between January 2006 and September 2011. Four raters independently appraised each CPG using the AGREE-II instrument and 2 reviewers independently extracted the data. Conflicts were resolved by discussion or the involvement of an additional reviewer. Eleven CPGs were identified. The overall quality ranged from 2.5 to 6 out of 7 on the AGREE-II tool. The highest scores were for “clarity of presentation” (44.4% −88.9%) and the lowest were for “rigour of development” (8.3%–30% for 9 CGPs). None of them clearly reported being newly developed or adapted. Only one reported having a patient representative in its development team. Systematic reviews were not consistently used and only 2 up-to-date Cochrane reviews were cited. Two CPGs graded some recommendations and related that to levels (but not quality) of evidence. The CPGs' recommendations on assessment and non-pharmacological management were fairly consistent. Guidelines varied in the selection of first-line treatment, adjustment of therapy and drug combinations. Important specific aspects of care (e.g. resistant hypertension) were ignored by 6/11 CPGs. The CPGs varied in methodological quality, suggesting that their implementation might not result in less variation of care or in better health-related outcomes.

**Conclusions/Significance:**

More efforts are needed to promote the realistic approach of localization or local adaptation of existing high-quality CPGs to the national context.

## Introduction

Globally, the prevalence of hypertension among adults aged 25 and over was approximately 40% in 2008 [1] and the total economic burden of hypertension in the United States was estimated at $73.4 billion in 2009 [2].

Better hypertension management leads to improved health outcomes. A large systematic review of 147 trial reports on the management of hypertension has shown that a reduction of 10 mm Hg in systolic blood pressure and 5 mm Hg in diastolic was associated with a 20% reduction of coronary heart disease and 32% reduction in stroke in one year [3]. And, the management of hypertension is cost-effective; treatment with medication results in improved health outcomes (higher quality-adjusted life-years; QALYs) [4]. However, awareness of hypertension, its treatment and control are far from adequate worldwide [5]–[7]. The variation in the multiple CPGs on hypertension published between 1997 and 2005 has been addressed in an earlier study [8] and it is clear that variation in the quality of guidelines exists for other conditions and is not unique to hypertension [9]–[13]. Of the CPGs used in 235 studies assessing the effectiveness and efficiency dissemination and implementation strategies, only 3% of guidelines used were based on good evidence [14].

The aim of this systematic review was to assess the quality and consistency of recommendations of recently-developed national and international CPGs on the diagnosis, assessment and the management of hypertension and, to determine the extent to which these CPGs are informed by Cochrane and non-Cochrane systematic reviews.

## Methods

This systematic review was completed based on a protocol with input from experts in hypertension and systematic review methodology, as recommended in the PRISMA Statement [15] (Table S1). The institutional review board was not obtained because there was no direct involvement with patients or bodily samples.

### Eligibility criteria

Multi-disciplinary CPGs endorsed by a national governmental or provider organization related to the diagnosis, assessment and management of hypertension were included. All subgroups of the population had to be examined to ensure that the CPGs cater for the needs of those with comorbidities in different settings; CPGs focused exclusively on hypertension among special groups (e.g. pregnancy, children, elderly, blacks or diabetes) or specific settings (e.g. primary care only or emergency management only) were excluded. To ensure that the most up-to-date CPGs were included, inclusion was limited to January 2006 onwards. Furthermore, only CPGs written in English were included.

### Information sources

Medical Subject Headings and text words related to hypertension and guidelines were used to search MEDLINE and EMBASE using the OVID interface from January 2006 to September 2011. The electronic database search was supplemented by searching websites and Google, as CPGs are not always cited in such databases. Specifically, the following websites were searched: Guidelines International Network (G-I-N; www.g-i-n.net), National Guidelines Clearinghouse (www.guideline.gov), Australia National Health and Medical Research Council (www.nhmrc.gov.au/guidelines/index.htm), National Institute for Health and Clinical Excellence (www.nice.org.uk) and Scottish Intercollegiate Guidelines Network (SIGN; www.sign.ac.uk), The word ‘hypertension’ was entered into the website search utility and the first 30 results were reviewed. Google was also searched using the keywords ‘hypertension’ and ‘guideline’ in a similar manner. To ensure all potentially relevant guidelines were identified, targeted searching by country was conducted in Google, the reference lists of included CPGs were scanned, and a list of the included guidelines were emailed to experts in the field to identify additional CPGs.

### Search

An experienced information specialist (LP) conducted all of the literature searches. The search strategy for the main electronic search (MEDLINE) is presented in Box S1; details on the EMBASE search are available upon request.

### Study selection

To ensure reliability, a training exercise was conducted prior to commencing the study selection process using a random sample of 25 citations. Two reviewers independently screened the search results for inclusion using a pre-defined relevance criteria form. The full-text article was obtained for potentially relevant CPGs and these were subsequently screened by two independent reviewers. Discrepancies at any stage were resolved by discussion or the involvement of a third reviewer.

### Data collection process and data items

A draft data extraction form was developed, piloted, and modified as necessary. Two reviewers independently extracted all of the data using the standardized data extraction form. Discrepancies were resolved by discussion or the involvement of a third reviewer.

All the relevant documents and websites of the selected CPGs were examined. The extracted data included CPG characteristics (e.g., year of dissemination, country/region, development team, funding organization), recommendations related to the diagnosis and assessment of hypertension, and recommendations related to the management of hypertension. The Appraisal of Guidelines Research and Evaluation (AGREE) II tool [16] was used by 4 reviewers independently to appraise the validity of each included CPGs. The 4 assessors also provided their judgments on the overall assessment, the possible risk of bias and recommendation for future use for each CPG that they appraised. Discrepancies were resolved by discussion or the involvement of a fifth reviewer. The agreement of the 4 raters in the “Rigour of Development” domain was explored using percentage of agreement. To measure inter-rater agreement, values for the eight items were collapsed from 7 to 3 values as follows: 1, 2, 3 as 1 to represent “disagree” and 5, 6 and 7 as 2 to represent “agree” and 4 becomes 3 as “neutral “. This analysis was conducted using the AgreeStat software [17].

The reference list of each of the selected CPGs was reviewed and the number of Cochrane and non-Cochrane systematic reviews in each was recorded. The end of search date for each CPG was checked to determine the available and relevant reviews prepared by the Cochrane Hypertension Group [18] by that date. For CPGs where the end-of-search date was not reported, the principal author was contacted. If there was no response from the author, it was assumed that search ended one year prior to publication of the CPG. Two reviewers independently screened all the abstracts of the reviews prepared by the Cochrane Hypertension Group to assess their relevance to the general management of primary hypertension.

### Synthesis of results

The included CPGs were summarized descriptively according to diagnosis, assessment and management recommendations. For each item, we noted whether the CPG recommended it, the level of evidence (which is based on the study design), and the quality of studies supporting/refuting the recommendation (determined when the reviewers critically appraised the studies). For diagnosis and assessment, the following categories were used: identification of cardiovascular risk factors, blood pressure measurement methods, medical history, physical examination, subclinical organ damage, and laboratory investigations. For management, the following categories were used: lifestyle modifications, initiation of therapy, type of therapy, adjustment of therapy, combination therapy, harms associated with the therapy, consideration of special groups (e.g., elderly, diabetics, renal dysfunction, pregnancy), follow-up, compliance, and specialist referral.

## Results

### Study selection

**Figure 1:**
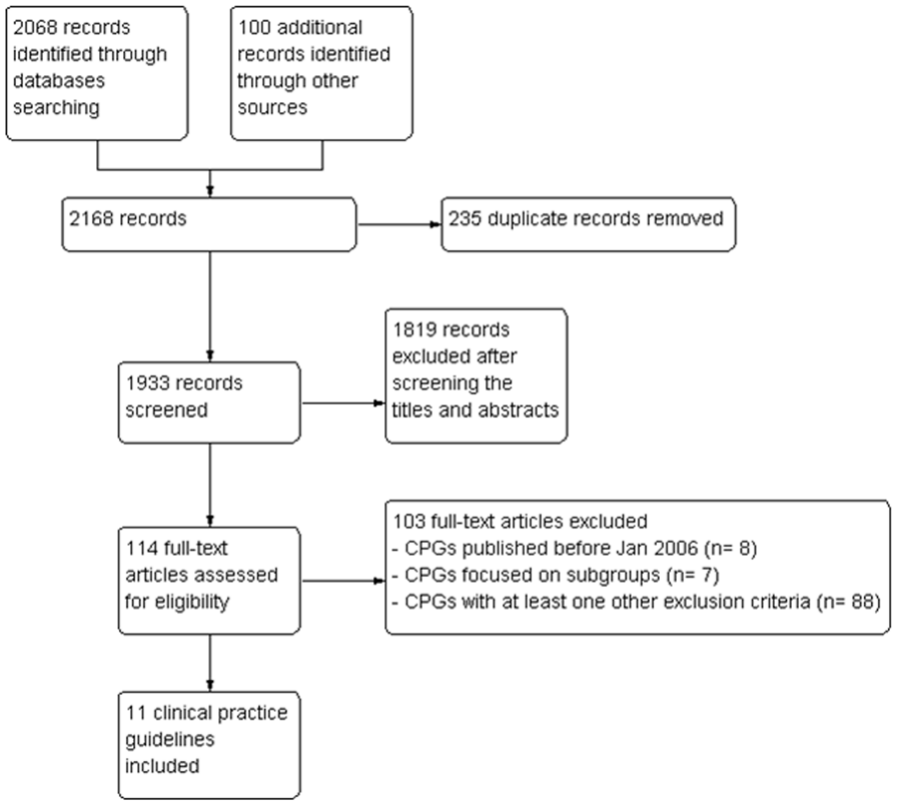
Flow chart using the PRISMA statement for the systematic review.

The search strategy retrieved 2168 citations, of which 114 were considered for full-text screening and 11 were included in the review (Figure 1). Two CPGs were multinational efforts to develop a unified hypertension CPG (EUR and LAT) [19]–[21]. The remaining hypertension CPGs were conducted in South Africa (SOA) [22], India (IND) [23], Poland (POL) [24], Malaysia (MAL) [25], Japan (JAP) [26], Australia (AUS) [27], Canada (CAN) [28], Saudi Arabia (SAU) [29] and the United Kingdom (NICE) [30].

### Clinical practice guideline characteristics

Table 1 displays the characteristics and methods related to CPG development. Two CPGs were new (POL and LAT); the rest were updates. All CPGs (except the SAU and IND CPGs) were retrieved through searching the medical literature databases. The SAU and IND CPGs were retrieved through the country-specific Google search.

**Table 1:** Characteristics and Methods Used For Developing the 11 Clinical Practice Guidelines.

Characteristics	SOA 2006	IND 2007	POL 2007	MAL 2008	EUR 2009	JAP 2009	LAT 2009	AUS 2010	CAN 2011	SAU 2011	NICE 2011
**Status of the CPG**											
New	No	No	Yes	No	No	No	Yes	No	No	No	No
Updated	Yes	Yes	No	Yes	Yes	Yes	No	Yes	Yes	Yes	Yes
**Level of development**											
National	Yes	Yes	Yes	Yes	No	Yes	No	Yes	Yes	Yes	Yes
Regional	No	No	No	No	Yes	No	Yes	No	No	No	No
**Organization behind the guideline**											
Professional organization (e.g. Societies)	Yes	Yes	Yes	Yes	Yes	Yes	Yes	Yes	Yes	Yes	Yes
Government	Yes	No	No	Yes	No	No	No	No	No	Yes	Yes
**Funding/Sponsorship**	No	NR	NR	Industry educational grant	NR	NR	NR	Professional Grants	Professional grants	Industry	NICE
**Developing team structure & affiliation described**											
Number of members	7	33	15	17	32	30	14	14	65	19	15
Affiliation described?	No	No	Yes	Yes	Yes	Yes	No	Yes	Yes	Yes	Yes
Specialty described?	No	No	No	Yes	Not clear	No	No	No	Yes	Yes	Yes
**Searching and selecting references**											
Search Strategy Described?	No	No	No	No	No	No	No	Yes	Yes	No	Yes
Total references cited	110	146	4	251	293	742	157	64	56	53	662
Total systematic reviews cited	6	0	0	19	19	36	5	6	6	8	12
Total Cochrane reviews cited	0	0	0	3	1*	8	1	0	0	0	3
Total cited/available** relevant Cochrane reviews from the Hypertension group only	0/11	0/15	0/15	0/17	Jan-32	0/32	0/32	0/34	0/39***	0/39***	2****/39
**Methods of deriving recommendations**											
Evidence linked, Formal consensus method	No	No	No	No	No	No	No	No	Yes	No	Yes
Evidence-linked, no description of method	No	No	No	Yes	No	No	No	Yes	No	No	No
Consensus method, no detailed description	Yes	Yes	No	No	No	No	Yes	No	No	No	No
Not described	No	No	Yes	No	Yes	Yes	No	No	No	Yes	No
**Implementation strategies described**	Yes	No	No	No	Yes, weak	No	No	Yes	Yes	Yes	Yes
Year of publication of the previous version of the CPG	2003	2001	-	2002	2007	2004	-	2009	2010	2007	2006
Next Update of the CPG	2012	NR	NR	2012	NR	NR	NR	NR	2012	NR	NR

NR: Not reported. SOA: South Africa; IND: India; POL: Poland; MAL: Malaysia; EUR: Europe; JAP: Japan; LAT: Latin America; AUS: Australia; CAN: Canada, SAU: Saudi Arabia and NICE (The UK's National Institute for Health and Clinical Excellence).

* The ESH Reappraisal in 2009 cited only 1 review (4 reviews were cited in 2007).

** Produced by the Hypertension Cochrane Review Group calculated for up to one year before the date of publication of the CPGs when the search date was not reported.

*** The total number of reviews available at that time was 41 but two reviews were excluded because they were judged as irrelevant.

**** The updated version of Murlow's review was published in 2008 but the 2000 version was the one cited.

The affiliation and/or the specialties of the developing group team members were not described in three CPGs (SOA, IND, LAT); the remaining guidelines provided some or a detailed description of the team. Two CPGs were funded by drug companies (SAU, MAL), three reported funding from professional organizations and provided a list of members with their declaration of interest (AUS, CAN, NICE), and the remainder did not disclose a funding source. In the CAN guideline, members with conflict of interest for certain recommendations were “recused” from voting. The size of the guideline development team varied from 7 to 65 members. Most of the CPGs (7/10) provided information on the affiliation of these members but only 3 (MAL,CAN and SAU) provided information on their specialties. Except for one CPG (NICE), 10/11 CPGs did not report including patient representatives in their guideline development team. Apart from the AUS, CAN and NICE CPGs, none of the guidelines reported a search strategy in their methods section. All CPGs (except for the IND and POL) cited some systematic reviews in the reference section. The number of systematic reviews cited ranged from 5 to 31. Five CPGs (SOA, IND, POL, AUS, CAN) did not refer to reviews from the Cochrane Collaboration developed by the Hypertension Review Group that were available at the time of guideline development. The JAP, SAU, MAL, LAT and NICE cited 8, 5,3, 1 and 3 Cochrane reviews, respectively. Of the reviews from the Cochrane Hypertension Group, one was cited by the EUR CPG [31] and 2 were cited by NICE CPG [32], [33]. Table 1 shows the numbers of available and relevant reviews from the Hypertension Group for each CPG that could have potentially been used by the guidelines' development teams. Some of the guidelines clearly reported that they referred to other international guidelines (SAU, LAT and IND) )but none of them reported being an adaptation of another CPG.

### AGREE-II appraisal results

In general, the guidelines received the lowest scores for rigour of development among all 6 AGREE domains (mean 27%, range: 8.3%–86.4%), whereas, they scored highest on clarity of presentation (mean 66.8%, range: 44.4%–88.9%). The CAN CPG scored the highest on rigour of development (Domain 3) and the NICE CPG scored the highest for the scope and purpose and editorial independence (Domains 1 and 6; Tables 2–3). The applicability (Domain 5) and stakeholder involvement (Domain 2) domains were scored consistently low across the CPGs (Tables 2–3). The overall quality of the CPGs ranged from 2.5 to 6 on a 7 point scale. With the exception of the CAN CPG, all guidelines were either not recommended for use or were recommended for use with modifications. The risk of bias (judged by the reviewers as an inverse overall assessment of the rigour of development domain) was lower in the CAN and NICE CPG and higher in the SOA, POL, EUR, LAT and the SAU CPGs. The degree of agreement among reviewers was tested using percentage of agreement for the rigour of development domain. The agreement varied across the guidelines from as high as 88% (CAN, POL), 73%,71%, 69%, 62% (SOA, JAP, LAT, MAL, respectively) to as low as 58%, 56%, 52%, 50%, 46% (IND, MAL, AUS, NICE, SAU, respectively)

**Table 2:** Domain Scores (%) for the 11 Clinical Practice Guidelines Using the AGREE-II Instrument.

	SOA 2006	IND 2007	POL 2007	MAL 2008	EUR 2009	JAP 2009	LAT 009	AUS 2010	CAN 2011	SAU 2011	NICE 2011
**DOMAIN 1. SCOPE AND PURPOSE**	47.2	44.4	25	65.3	36.1	20.8	61.1	22.2	75	44	83
**DOMAIN 2. STAKEHOLDER INVOLVEMENT**	37.5	13.9	12.5	45.8	27.7	18	41.6	38.9	75	49	74
**DOMAIN 3. RIGOUR OF DEVELOPMENT**	13.5	21.8	8.3	26.5	23.4	18.75	15.6	27.1	86.4	30	62
**DOMAIN 4. CLARITY OF PRESENTATION**	55.5	50	75	69.4	69.4	62.5	44.4	88.9	88.9	64	55
**DOMAIN 5. APPLICABILITY**	38.5	17.7	16.6	42.7	21.8	14.6	30.2	59.3	59.3	46	72
**DOMAIN 6. EDITORIAL INDEPENDENCE**	39.6	16.6	4.1	68.75	64.6	29.1	35.4	64.6	75	38	88

SOA: South Africa; IND: India; POL: Poland; MAL: Malaysia; EUR: Europe; JAP: Japan; LAT: Latin America; AUS: Australia; CAN: Canada; SAU: Saudi Arabia and NICE: UK's National Institute for Health and Clinical Excellence.

**Table 3:** Quality of the 11 Hypertension Clinical Practice Guidelines for the six domains of the AGREE-II Instrument (D1–D6) and the Overall Impression of the 4 Assessors.

	D1			D2			D3								D4			D5				D6		Overall*	Risk of Bias**	Recommend CPG for Use***
*Item #*	*1*	*2*	*3*	*4*	*5*	*6*	*7*	*8*	*9*	*10*	*11*	*12*	*13*	*14*	*15*	*16*	*17*	*18*	*19*	*20*	*#*	*22*	*23*			
**SOA 2006**	5	3	4	4	1	5	1	1	1	2	2	2	4	3	5	6	3	3	4	3	3	5	2	3	+++	No
**IND 2007**	4	3	4	2	2	2	2	2	1	1	2	2	5	2	4	5	3	2	2	2	2	3	1	2.5	++	No
**POL 2007**	2	1	4	3	1	1	1	1	2	1	3	2	1	1	6	6	5	1	2	1	3	1	1	3	+++	No
**MAL 2008**	5	5	5	5	2	5	2	1	1	1	4	4	4	4	5	6	5	3	4	4	4	6	5	3	++	Unsure
**EUR 2009**	3	3	3	4	1	3	2	3	4	1	4	2	3	1	5	6	5	5	2	2	1	4	6	3.5	+++	No
**JAP 2009**	2	2	3	3	1	2	2	2	2	2	4	3	2	1	4	6	5	2	2	2	1	4	2	4	++	Unsure
**LAT 2009**	6	4	5	3	3	5	2	1	1	2	3	2	3	3	4	4	3	3	2	3	4	4	2	3	+++	Unsure
**AUS 2010**	2	3	3	4	4	2	3	1	1	3	4	2	6	2	6	6	7	5	6	3	5	3	7	4.5	++	Yes, with modifications
**CAN 2011**	5	6	6	7	5	6	7	7	6	7	6	6	5	7	7	7	6	4	6	4	4	5	6	6	+	Yes
**SAU 2011**	4	4	4	5	3	4	3	3	2	3	3	3	2	4	5	6	4	5	4	3	3	4	3	3.5	+++	No
**NICE 2011**	6	6	6	6	5	6	6	6	6	6	5	5	4	5	6	6	6	5	6	6	6	7	6	6	+	Yes, with modifications

D1 : Scope & purpose, D2: Stakeholder involvement, D3: Rigor of involvement, D4: Clarity of presentation, D5: Applicability, D6: editorial independence.

All the 23 items of the AGREE-II instrument are rated on a 7-point scale where a score of 1 is given when there is no information that is relevant to the item or if the concept is very poorly reported; a score of 7 is given if the quality of reporting is exceptional and where the full criteria and considerations articulated in the AGREE-II User's Manual have been met; and a score between 2 and 6 is assigned when the reporting of the AGREE II item does not meet the full criteria or considerations. Scores increase as more criteria are met and considerations addressed. In other words, the higher the score, the better the quality of the CPG item.

SOA: South Africa; IND: India; POL: Poland; MAL: Malaysia; EUR: Europe; JAP: Japan; LAT: Latin America; AUS: Australia; CAN: Canada; SAU: Saudi Arabia and NICE: UK's National Institute for Health and Clinical Excellence).

* Although the scoring is done in integers, the numbers in this column represent the averages of the scoring done by 4 assessors.

** Risk of bias: +++ high, ++ intermediate, + low.

*** This is based on the subjective assessment made individually by each of the 4 assessors in response to: “Do you recommend this CPG for use?”

Only two guidelines linked their grade of recommendations to the level of evidence (MAL, CAN), yet they did not elaborate on the quality of studies contributing to the recommendations (Table 4). Agreement between these CPGs on the grade of recommendations was not observed. For example, the advice on exercise was graded A in MAL and D in CAN. The NICE CPG provided the evidence tables for their recommendations and the SAU CPG reported the level of evidence for some recommendations but none of them reported the strength of recommendations. The other 7 CPGs did not disclose the level of evidence or how their recommendations were decided upon.

**Table 4:** Strength of the recommendations stated in the Malaysian and Canadian Clinical Practice Guidelines.*

Recommendations	Strength of recommendation	
	MAL 2008	CAN 2011
Recommendations to attain normal body mass index	C	B
An intake of <100 mmol of sodium daily	A	B
Advice to restrict intake of alcohol	C	B
General advice on exercise	A	D
Adapting healthy DASH diet	A	B
Smoking Cessation	C	Not graded
Recommendations to use ACEI in presence of microalbuminuria	A	A
Use of ARB if ACEI is not tolerated	A	B
Recommendation for diuretics or calcium channel blockers as alternative therapy in diabetic hypertensive patients	A	A
Combination of ACEIs and ARBs in patients with hypertension and no diabetic renal disease	A	B

* None of the other CPGs stated their strength of recommendations.

MAL: Malaysia; CAN: Canada.

### Clinical practice guideline recommendations

#### Definition

Most CPGs considered high normal blood pressure to range from 120–129 systolic blood pressure (SBP) or 80–84 for diastolic blood pressure (DBP) (Table 5). The exception was the IND and NICE CPGs, which defined hypertension using a higher cut off points. The CAN CPG did not use cut off points for hypertension.

**Table 5:** Recommendations from Clinical Practice Guidelines About Diagnosis and Assessment of Patients with Hypertension.

ITEM	SOA 2006	IND 2007	POL 2007	MAL 2008	EUR 2009	JAP 2009	LAT 2009	AUS 2010	CAN 2011	SAU 2011	NICE 2011
**Definition of Hypertension**											
Normal: SBP (120–129) or DBP(80–84), if different, state	√	SBP<130 DBP<85	√	SBP<120, DBP<80	√	<125/80	√	√	NR	SBP<120 and DBP<80	Clinic <140/90 mmHg or HBPM/ABPM <135/85
High normal: SBP (130–139) or DBP (85–89) if different, state	√	√	√	Pre-HTN SBP 120–139 mmHg, DBP 80–89 mmHg	√	√	√	SBP (120–139) or DBP (80–89)	√	Pre-HTN 120–139, and/or 80–89	X
Mild (G1): SBP (140–159) or DBP (90–99)	√	√	√	√	√	√	√	√	X	√	Clinic ≥140/90 mmHg and HBPM/ABPM ≥135/85
Moderate (G2): SBP (160–179) or DBP (100–109)	√	√	√	√	√	√	√	√	X	√	Clinic ≥160/100 mmHg and ABPM/HBPM ≥150/95 mmHg
Severe (G3): SBP>180 or DBP>110	√	√	√	√	√	√	√	√	X	≥180 and/or ≥110	Clinic SBP ≥180/110 mmHg
Isolated systolic hypertension: SBP>140 and DBP<90 if different, state	NR	√	√	√	√	√	√	√	NR	NR	SBP ≥160 mmHg
Isolated systolic hypertension+widened pulse pressure: SBP>160 and DBP<70	NR	√	√	√	√	√	√	√	NR	NR	NR
**Cardiovascular Risk Assessment**											
**Data elements recommended for cardiovascular risk stratification**											
SBP/DBP	√	NR	√	NR	√	√	√	√	√	√	√
Smoking	√	√	√	√	√	√	√	√	√	√	√
Dyslipidaemia	√	√	√	√	√	√	√	√	√	√	√
Diabetes	√	√	√	√	√	√	√	√	√	√	√
**Subclinical organ damage**											
LVH on ECG/ECHO	√	√	√	√	√	√	√	√	√	√	√
Microalbuminuria: ECR 3–30 mg/mmol	√	1.2–2 mg/dl	Recommended but cut-off not reported	≥ 2.0 mg/mmol (males) or ≥2.5 mg/mmol (females) on spot urine screening test OR 24-hour urinary albumin excretion rate ≥20 µg/minute	30–300 mg/24 hours	Recommended but cut-off not reported	Recommended but cut-off not reported	≥2.0 mg/mmol (males) or ≥2.5 mg/mmol (females) on spot urine screening test OR 24-hour urinary albumin excretion rate ≥20 µg/minute	Recommended but cut-off not reported	NR	Albuminuria stated but no cut-off reported
**CKD**											
Elevated creatinine: Men 115–133, Women 107–124 µmol/l	√	elevated serum creatinine 1.2–2.0 mg/dl	Recommended but no cut-off	x	√	√	CR>1.3 mg/dL	X	x	√	Reported with no cut-off stated
Proteinurea protein/creatinine ratio ≥30 mg/mmol on spot urine test or urine protein >300 mg/day on timed urine sample	√	x	Urinary albumin/creatinine ratio but the ratio is not stated	Urinary protein >500 mg/24 hr or albumin to creatinine ratio [ACR] >30 mg/mmol	Albumin-creatinine ratio: >or = 22 (M); or 31 (W) mg/g creatinine	√	x	√	√	NR	Reported with no cut-off stated
eGFR <60 mL/minute/1.73 m	×	√	√	√	√	√	eGFR <30 ml/min/1.73 m	√	√	NR	Reported with no cut-off stated
***Subclinical organ damage: Vascular disease***											
Atherosclerotic plaque (aorta, carotid, coronary, femoral and iliac arteries) evident on US or radiology	Not stated clearly	√	√	√	√	√	√	√	√	√	√
Hypertensive retinopathy (grade II or greater)	√	√	×	√	√	√	grade III/IV	√	√	√	√
Stratification: Low; Moderate; High/Very High added risk	normal, high normal, mild, moderate, severe	√	normal, high normal, Grade 1, 2 and 3)	√	√	√	low, intermediate and high	Low, Mod, High	NR	√	NR
**BP measurement**											
Office: 140/90	√	√	√	√	√	√	√	√	√	√	√
Home: 135/85	√	√	×	√	√	√	√	×	√	√	√
Ambulatory: 120/70 (mean night); 135/85 (mean day); 130/80 (24-hour) * Suggested for selected cases	√*	√*	×	√*	√*	√	√*	√*	√	√*	√*
Reason(s) for Home and self-monitoring	For select groups	For White-Coat HTn only	NR	White coat HTN Monitoring	White-coat HTN Monitoring Dx of resistant HTN	More accurate Dx Masked and white-coat HTN Improves adherence	Masked and white-coat HTN and FU	Masked and white-coat HTN and FU	For white-coat HTN	White-coat HTN Monitoring Dx of resistant HTN	confirm diagnosis, white-coat HTN
List of devices provided	√	NR	NR	√	√	√	√	NR	NR	√	√$
Both arms * First visits only	√*	√*	NR	√	√*	NR	√	√*	√*	√*	√*
**Family History**											
Early CVD: Men aged <55 years and Women aged <65 years	√	√	√	√	√	√	Not clear	√	√	√	√
High blood pressure	×	√	NR	√	√	√	NR	√	NR	√	√
Obesity	×	√	×	√	×	√	√	×	×	√	×
Stroke	×	√	×	√	√	√	√	√	×	√	×
Dyslipidaemia	×	√	×	√	√	x	×	√	×	×	×
Diabetes	×	√	×	√	√	√	NR	√	×	×	×
**Clinical History**											
CAD	√	√	√	√	√	√	√	√	√	√	√
Heart Failure	√	√	√	√	√	√	√	√	√	√	√
CKD	√	√	√	√	√	√	√	√	√	√	√
Stroke or TIA	√	√	NR	√	√	√	√	√	√	√	√
Peripheral vascular disease	√	√	NR	√	√	√	√	√	√	√	√
Retinopathy	√	√	NR	√	√	√	√	√	√	√	√
Aortic disease	×	√	NR	√	√	√	√	√	×	√	√
Hypercholesterolaemia: Serum TC>7.5 mmol/L	√	√	√	√	√	√	×	√	√	√	√
Previous medications	×	√	NR	√	√	√	√	√	√	√	√
Other significant conditions (asthma, sleep apnea, COPD)	×	×	NR	√	√	√	×	√	×	√	√
Modifiable lifestyle risk factors	√	√	√	√	√	√	NR	√	√	√	√
History of hypokalaemia or suggestive symptoms	×	×	NR	√	×	NR	×	√	√	√	√
Other	-	Smoking, gout, sexual dysfunction, Dietary (Salt, Alcohol, Caffeine)	Smoking and Gout	personal, psychosocial and environmental factors	Smoking, dietary, obesity, physical exercise	-	-	psychosocial and environmental factor	-	Growth retardation	Symptoms of identifiable cause s of HTN
**Physical Examination**											
Cardiovascular	×	√	√	√	√	√	√	√	√	√	√
ECG	√	√	NR	√	√	√	√	√	√	√	√
Obesity( Waist-to-hip ratio or BMI)	√	√	√	√	√	√	√	√	√	√	√
Other physical examination	Body weight	-	-	Abnormalities of optic fundi evidence of abnormalities of the endocrine system (e.g. Cushing's syndrome, thyroid disease)	-	-	associated risk factors and possible complications such as peripheral edema, angina pectoris, dyspnea, headache, ectopic heart beats	ABI	-	Typical cushingoid appearance	Signs of secondary causes
**Searching for subclinical organ damage**											
Heart: LVH	√	√	√	√	√	√	√	√	√	√	√
Blood vessels: Peripheral arterial disease	√	√	NR	√	√	√	√	√	√	√	√
Blood vessels: Aortic disease	NR	NR	NR	√	NR	√	NR	√	NR	NR	√
Kidney: CKD	√	√	√	√	√	√	√	√	√	√	√
Kidney: Other	elevated creatinine	Albumin/CR Ration	-	-	-	-	ultrasound/Doppler for renal arterial stenosis or kidney alterations	Diabetic nephropathy, Glomerulonephritis, Hypertensive kidney disease.	-	Diabetic nephropathy	-
Fundoscopy: Haemorrhages OR Exudates or Papilloedema	√	√	NR	√	√	√	NR	√	√	√	√
Brain: Stroke or TIA	√	√	√	√	√	√	√	√	√	√	√
**Lab investigations**											
Urine dipstick for blood, protein sugar	√	√	√	√	√	√	√	√	√	√	√
Microalbuminurea	√	√	√	√	√	√	√	√	√	√	√
Blood tests: FBG, random total cholesterol, creatinine, potassium.	√	√	√	√	√	√	√	√	√	√	√
ECG	√	√	NR	√	√	√	√	√	√	√	√
C-reactive protein > l mg/dl	NR	√	NR	NR	NR	√	NR	NR	NR	NR	NR
Other Investigations	-	Echo uric acid	Carotid-femoral pulse wave velocity glomerular filtration rate	ABI, ECD, Plasma aldosterone/renin ratio	-	-	Thyroid function test, LFT	ECD, Plasma aldosterone/renin ratio24-H urinary catecholamine, RAU evidence of abnormalities of the endocrine system thyroid disease)	Screening for hyperaldosteronism (hypokalemia) or hypokalaemia Screening pheochromocytoma renovascular hypertension Captopril-enhanced radioisotope renal scan Doppler sonography, magnetic resonance angiography and CT- angiography (for those with normal renal function)	CBC Uric acid TSH, free T4 CXR abdominal US Echo Significantly 24-H urinary catecholamines Overnight dexamethasone suppression testing Plasma aldosterone/renin ratio	total/HDL cholesterol levels, TSH, polysomnograph

NR: Not reported, √: Recommended, ×: Not Recommended; SOA: South Africa; IND: India; POL: Poland; MAL: Malaysia; EUR: Europe; JAP: Japan; LAT: Latin America; AUS: Australia; CAN: Canada; SAU: Saudi Arabia and NICE (The UK's National Institute for Health and Clinical Excellence).

$ not endorsed by NICE. ABI: ankle-brachial index ECD: Echo Carotid Doppler, RAU: Renal artery duplex ultrasound.

“White coat syndrome” (i.e., the propensity for patients to have higher blood pressure when measured by a clinician) was addressed using self-measured blood pressure in 9/11 CPGs. The devices used in self-monitoring were described in 7 CPGs (SOA, MAL, EUR, JAP, AUS, CAN and NICE). The distinction between different settings (office, home and ambulatory) for measuring blood pressure and identifying patients as having hypertension was made in all CPGs except two (POL, AUS).

### Cardiovascular Risk

All CPGs recommended assessing hypertension in relation to other cardiovascular risk factors during patient assessment (Table 5).

### Family and Clinical history

The clinical assessment included asking patients about their family history of hypertension (IND, MAL, EUR, JAP, LAT, SAU, NICE), stroke (IND, MAL, EUR, JAP, LAT, AUS, and SAU), dyslipidemia (IND, MAL, EUR and AUS) and diabetes (IND, MAL, EUR, JAP and AUS). All CPGs recommended inquiring about previous coronary artery disease, heart failure and chronic kidney disease (Table 5). All but one CPG (POL) recommended asking about past history of stroke and existing peripheral artery disease and retinopathy.

### Physical examination searching for subclinical organ damage

All CPGs recommended assessing the patient's body mass index. Similarly, all addressed modifiable lifestyle risk factors, except for one CPG (LAT) (Table 5). All except for one CPG (POL) considered ECG as a necessary component of the physical examination. All recommended fundoscopy, except for the POL and LAT CPGs.

### Laboratory testing

All CPG suggested assessing fasting blood glucose, fasting blood cholesterol, creatinine, potassium and urine dipstick testing for glucose, blood (hematuria), protein and albumin (Table 5). Only two CPGs (IND and JAP) recommended assessing C-reactive protein as part of the workup for patients with hypertension.

### Recommendations for the management of hypertension:

Findings from guidelines about the management of hypertension are presented in Table 6. All guidelines advocated similar life style changes as a cornerstone in the management of hypertension. Minor differences included recommendation of dietary supplements, increase of potassium intake, exercise, and stress and emotional management.

**Table 6:** Recommendations from Clinical Practice Guidelines about Managing Patients with Hypertension.

ITEM	SOA 2006	IND 2007	POL 2007	MAL 2008	EUR 2009	JAP 2009	LAT 2009	AUS 2010	CAN 2011	SAU 2011	NICE 2011
**Advice about Lifestyle changes**											
Maintain weight	√	√	√	√	√	√	√	√	√	√	√
Lower sodium intake	√	√	√	√	√	√	√	√	√	√	√
Limit alcohol	√	√	√	√	√	√	√	√	√	NR	√
Follow nutrition guidelines	√	√	√	√	√	√	√	√	√	√	√
Limit sugar intake	√	NR	√	NR	NR	NR	NR	√	√	NR	NR
Lower fat intake	√	√	√	√	√	√	√	NR	√	√	√
moderate-intensity exercise for at least 30 minutes on most or preferably all days of the week	NR	NR	NR	√	√	√	√	NR	√	NR	√
Stop smoking	√	√	√	√	√	√	√	√	√	√	√
Other											
Dietary supplements	NR	NR	NR	√	NR	NR	NR	NR	NR	NR	NR
Increasing K	NR	NR	NR	NR	NR	NR	√	NR	√	NR	NR
Stress management	NR	NR	NR	√	NR	√	NR	NR	√	NR	√
**When to initiate Therapy?**											
Low added risk despite a period of 6–12 months of lifestyle modification and observation	√	3 months cut-off	NR	√^1^	√	3 months cut-off	√	√	√^2^	√	NR
Moderate added risk despite a period of 3–6 months of lifestyle modification and observation	√	2–3 months cut-off	NR	√^3^	√	1 month cut-off	√	√	√^4^	√	NR
High or very high added risk	√	√	√	√^5^	√	√	√	√	√	√	√
**How to initiate drug therapy?**											
**Step 1** Use a low-dose diuretic as initial therapy	√	√	NR	NR	NR	NR	NR	NR	NR	√	NR
use agent from any of the 5 classes (A,B,C,D) as first line	NR	√	√	√	√	√	√	√	√	√	√^6^
**Step 2:** Consider costs, other conditions contraindications and if OK prescribe ACE-Is and CCBs	√	√	√	√	√	√	√	√	√	√	√
Other second-line medication from the 5 classes	√	√	√	√	√	√	√	√	√	√	√
**Step 3:** Other third-line medication?	Add A or C	A or B+C+D	A diuretic should be one of them	Combination of therapies	√	Add a third agent	√	√	Yes if not controlled	Renin inhibitors	A+C+D
**Adjustment of therapy**											
**Strategies:**											
Increase dose of 1st agent	NR	NR	NR	√	NR	√	NR	NR	NR	NR	√
Substitute with another agent	NR	√	NR	√	NR	√	NR	NR	NR	NR	NR
Add another agent	√	√	NR	√	√	√	NR	NR	NR	NR	√
Other Strategies	NR	intensify life style	long acting mono-therapy	NR	NR	give drug twice daily	NR	NR	Changes in nocturnal BP	NR	NR
**Choice of anti-hypertension therapy**											
Start with mono-therapy and move to combo therapy	√	√	√	√	√	√	not clear	√	√^7^	√	√
and/or two drug combination as initial	NR	√	NR	NR	NR	NR	NR	NR	NR	√^7^	NR
**Recommendations about combination therapy**											
Which drug combination?	D+BB	various	various	various	Various	Various	Not clear	A+C	various	A+C	A+C
**Considerations for special groups**											
Elderly	√	√	NR	√	√	√	√	√	NR	√	√
Diabetics	√	√	√	√	√	√	√	√	√	√	NR
Proteinuria	√	√	NR	√	√	√	√	√	√	√	√
Renal insufficiency	√	NR	NR	√	NR	√	√	NR	√	√	NR
renal failure	NR	√	√	NR	√	√	√	NR	NR	NR	√^8^
Bilateral artery stenosis	NR	NR	NR	√	NR	√	NR	NR	NR	NR	NR
Heart Failure	√	√	√	√	√	√	√	√	√	√	√
Post MI	√	√	√	√	√	√	√	√	√	√	NR
Angina	√	√	√	√	√	√	√	√	√	√	NR
Peripheral vascular disease	√	√	√	NR	√	√	√	NR	NR	√	NR
Carotid atherosclerosis	√	NR	NR	NR	√	√	NR	NR	NR	NR	NR
CCB for Supraventricular tachycardia	√	NR	NR	NR	√	√	√	NR	NR	√	NR
Left ventricular dysfunction/LVH	√	NR	√	√	√	√	√	NR	√	√	√
Tachyarrhythmias	√	√	NR	√	√	NR	√	√	√	√	NR
COPD	√	√	√	√	√	√	NR	√	NR	√	NR
Pregnancy	√	√	√	√	√	√	√	√	NR	√	NR
Metabolic Syndrome	NR	√	√	NR	√	√	√	NR	NR	√	NR
Resistant Hypertension	√	NR	NR	NR	√	√	NR	NR	NR	√	√
**HTN Emergencies**											
Hospitalization and IV drugs	√	√	NR	√	NR	√	√	NR	NR	√	NR
**Recommendations on managing associated risk factors**											
Antiplatelet therapy^8^	√	√	√	√	√	√	√	√	√	√	√^13^
Lipid Lowering agent^8^	NR	√	√	√^9^	√	√	√	√	√	√	√^13^
Glycemic control	NR	√^10^	NR	NR	√^11^	NR	√^12^	NR	NR	NR	NR
Frequency of follow up											
Frequency of follow up during stabilization phase	NR	NR	NR	NR	NR	NR	NR	Every 6 weeks or as needed	NR	Monthly or according to risk	NR
Frequency of follow up for patients with stabilized hypertension	√^14^	√^15^	NR	√^14^	NR	NR	NR	√^16^	NR	√^15^	√^17^
Assessment of compliance discussed	√	NR	NR	√	√	√	NR	NR	√	√	√
Strategies to improve adherence discussed	√	NR	NR	√	NR	√	NR	√	√	√	√
“When to Refer?” discussed	√	√	NR	√	NR	√	√	√	√^18^	√	√

SOA: South Africa; IND: India; POL: Poland; MAL: Malaysia; EUR: Europe; JAP: Japan; LAT: Latin America; AUS: Australia; CAN: Canada and SAU: Saudi Arabia and NICE (The UK's National Institute for Health and Clinical Excellence). NR: not reported. A: angiotensin converting enzyme inhibitor (ACEI), or angiotensin receptor blockers (ARB), C: calcium channel blocker (CCB), D: Diuretic.

1 And if SBP>150 and or DBP>95- treat.

2 If the SBP> or  = 140 mm Hg or DBP> or  = 90 mm Hg across 5 visits.

3 if SBP = 120–159 mmHg AND/OR DBP = 80–99 mmHg.

4 If, at visit 2 within one month, SBP is > or  = 140 mm Hg and/or DBP is > or  = 90 mm Hg.

5 If SBP = 120–159 mmHg AND/OR DBP = 80–99 mmHg with high risk or if SBP 160 mmHg AND/OR DBP 100 mmHg regardless of risk.

6 NICE CPG favored A for those below 55 years and C, D, for those aged 55 years or older and for black patients.

7 Yes if SBP>10 mmHg above target.

8 Recommended in at least certain high risk groups.

9 Recommended for those with atherosclerotic renal artery stenosis only.

10 No target level stated.

11 A1c<6.5 mmol/L.

12 A1c between 6.5–7% in patients with HTN, DM and nephropathy.

13 Referred to previous guideline version.

14 Every 3–6 months;

15 Every 3 months for high risk patients and every 6 months for low risk patients;

16 Every 3 months for the first year then 6-monthly thereafter;

17 Once a year.

18 For pheochromocytoma cases only.

All CPGs emphasized the need to stop smoking, maintaining weight, following nutritional guidelines, lowering sodium intake, limiting alcohol intake (except for SAU) and lowering fat intake (except for AUS) for hypertensive patients.

Most guidelines recommended the same criteria for initiating drug therapy; minor differences were noted regarding the duration of a life style modification trial before starting drug therapy. All CPGs recommended starting antihypertensive therapy without delay for patients with high blood pressure or high cardiovascular risk defined by most guidelines (except for the AUS CPG) as ≥20% risk of developing a cardiovascular event over 10 years. The AUS CPGs defined high risk as ≥15% risk of developing an event over 5 years.

Most CPGs recommended use of any of the 5 classes of antihypertensive drugs (angiotensin converting enzymes inhibitors, angiotensin receptor blockers, beta-blocker, calcium channel blockers or diuretics) as first line therapy. However, low-dose diuretics were preferred by the IND and SAU CPGs and were exclusively recommended by the SOA CPGs. The CPGs also differed in their strategies of adjustment of therapy. Most recommended adding another drug if the blood pressure is not adequately controlled (10/11); others suggested substituting with another drug (3/11) and/or increasing the dose of the first agent (3/11). Recommendations about drug combinations were variable across guidelines. Selection of therapeutic agents for compelling indications such as established cardiovascular disease or diabetes were similar, yet there were some differences in relative or absolute contraindication definitions. Only five CPGs discussed managing resistant hypertension and five CPGs did not discuss hypertensive emergencies. Controlling associated risk factors by the use of antiplatelet therapy, statins and/or glycemic control were addressed in 11/11, 10/11 and 3/11 CPGs respectively.

### Follow up, compliance, adherence strategies and referral

Only two CPGs (AUS and SAU) addressed how often patients should be seen during the stabilization phase. The AUS suggested that this should occur every 6 weeks or as indicated (which could be few days to 2 months). The SAU suggested monthly visits. Six CPGs suggested one of 2 plans for follow up of patients with stable BP; either to follow all patients every 3–6 months or to follow high risk (20% risk or higher) patients three monthly and low risk patients six monthly. The methods for assessing compliance with medication and strategies to improve adherence were discussed in 7/11 CPGs. The indications for referral to other specialties were discussed in 9/11 CPGs

## Discussion

Most of the CPGs clearly presented their recommendations. However methodological gaps exist across the guidelines that should be addressed including clarifying the scope and purpose, ensuring representation of all stakeholders including consumers, developing guidelines with scientific rigour, supporting implementation of the recommendations and declaring the presence or absence of editorial independence. These results are similar to a recent review of 42 reviews of guidelines (a total of 626 CPGs on a variety of topics) published between 1980 and 2007, which showed that despite some increase in quality of CPGs over time, the average quality scores as measured with the AGREE Instrument have remained moderate (43% for ‘Rigour of Development’) to low (35% for ‘Stakeholder Involvement’, 30% for ‘Editorial Independence’ and 20% for ‘Applicability’) [34].

In general, the recommendations of the CPGs on diagnosis, assessment and non-pharmacological management were consistent despite scoring poorly in their rigour of development. It is difficult to tell whether this happened because there was no evidence to guide or because the authors did not search and make use of the best available evidence. This finding is similar to that of Burgers, et al, who reviewed 15 CPGs for patients with diabetes from 13 countries [35]. They found an international consensus in the recommendations despite the variation in cited evidence and preferential citation of evidence in each CPG. The influence of professional bodies such as the American Diabetes Association was suggested an important factor in explaining international consensus. He concluded that globalization of recommended management of diabetes was not a simple consequence of the globalization of research evidence.

For example, all the CPGs have embraced the concept of traditional or global cardiovascular risk assessment as a method for stratifying treatment which is presumably an evidence-based move, yet the level of evidence for this recommendation was not reported. Keeping in mind the recent concerns as that the current methods for assessing risk may ignore some patient characteristics [36], [37], that short-term assessment of cardiovascular risk may not translate directly into life-time risk estimates [38], the recent calls for improving cardiovascular risk assessment [39], the finding that externally validated tools for cardiovascular risk assessment may not fit well with certain populations with different baseline risks [40] and that there is no consensus among the guidelines for assessing cardiovascular risk in healthy checks on their approach and screening tests [41], users of the CPGs may decide not to implement this recommendation.

Office blood pressure measurement was recommended as the mainstay for the diagnosis and/or monitoring of hypertension with ambulatory or home self monitoring being recommended for a selected group of patients in 10/11 CPGs. Although this pragmatic approach is attractive and may have many merits, this recommendation was linked to weak evidence in the CAN and MAL CPGs. A recent systematic review found that neither clinic nor home measurement had sufficient sensitivity or specificity (compared to ambulatory-monitoring) to be recommended alone as a diagnostic test. [42].

Across the CPGs, major differences were related to the pharmacologic management of hypertension, namely, the selection of first-line treatment, adjustment of therapy and drug combinations. Even when CPG developers claimed that they related their grade of recommendations to the level of evidence, recommendations were not graded or were inconsistent. This variation may be related to the developers' search strategy, the process of selecting the scientific evidence and the way the recommendations were formulated. [43].

As is the finding of earlier analysis of multiple CPGs on various condition, we found that guideline developers did not consistently use systematic reviews [35]. Only two up-to-date reviews from the Cochrane Hypertension Group [31] [33] were cited in the guidelines we reviewed. This finding is consistent with a recent analysis of 106 NICE guidelines, which showed that one fifth of the CPGs referred to no Cochrane citations and two fifths referred to only 1–5 Cochrane reviews [44] although the majority were felt to directly address guideline questions. It is surprising that despite the increased production of Cochrane reviews, recent CPGs barely referred to relevant reviews. The reasons for this need to be explored and the Cochrane Collaboration need to consider the practical means for increasing the uptake by guidelines developers.

### Limitations of this review

First, only CPGs that were written in English were included; high-quality CPGs written exclusively in other languages might have been missed. It has been shown that restricting the search for systematic reviews to English language only did not affect the quality of most reviews [45]

Second, only the AGREE-II instrument was used in assessing the quality of CPGs. Other instruments, such as the recently-published 4-item Global Rating Scale (GRS) may be used in addition to the AGREE-II instrument. A comparison of both instruments has shown that the GRS is less sensitive in detecting differences in guideline quality but it could predict important outcome measures related to guideline adoption [46]. Third, our search was limited to January 2006 to September 2011 because it is believed that CPGs should be assessed for validity every 3 years [47], [48]. As a result, well-known guidelines such as the US Seventh Report of the Joint National Committee on Prevention, Detection, Evaluation, and Treatment of High Blood Pressure (JNC 7) [49] and the World Health Organization/International Society of Hypertension guidelines [50] were excluded because they were published in 2003 and are likely to be out-of-date.

### Future steps

Despite these limitations, it is clear that more efforts are needed to improve the quality of the developed CPGs at the national or continental levels and to keep them up-to-date. With such variation and deficiencies in the methodological quality of CPGs, there is no guarantee that the recommendations would result in better health-related outcomes for patients with hypertension. Guidelines for developing high-quality evidence-based guidelines' have been established by various organizations [51]–[55].

Given the time-intensive and resource-intensive nature of CPG development, local adaptation of existing high-quality CPGs to the national context might be a more realistic approach to developing national or continental CPGs to avoid duplication of efforts [56]. Use of the ADAPTE framework [57] may be considered by local and national implementation teams and guideline developers and [58] de novo guideline development would only be needed if no high quality guideline exists for a given topic.

## Supporting Information

Box S1 **Medline Search Strategy.**
(DOCX)

Table S1 **PRISMA 2009 Checklist for the systematic review of 11 recent hypertension clinical practice guidelines.**
(DOC)
