# Supplementary material for: A Systematic Review of Recent Clinical Practice Guidelines on the Diagnosis, Assessment and Management of Hypertension
Source: PLoS One. 2013 Jan 17;8(1):e53744. doi: 10.1371/journal.pone.0053744 (PMC3547930; doi:10.1371/journal.pone.0053744)
Supplement: Box S1 — Medline Search Strategy. (DOCX) [file pone.0053744.s001.docx]

**Box S1: MEDLINE SEARCH STRATEGY**

Database: Ovid MEDLINE(R) <1950 to September Week 35 2011>

Search Strategy:

--------------------------------------------------------------------------------

1 guideline.pt.

2 practice guideline.pt.

3 Health Planning Guidelines/

4 Consensus Development Conference/

5 (guideline or guidelines).m_titl.

6 *Clinical Protocols/

7 or/1-6

8 exp Hypertension/

9 "high blood pressure".tw.

10 hypertension.tw.

11 or/8-10

12 7 and 11

13 limit 12 to yr="2005 -Current"
